# Targeting the Kaposi’s sarcoma-associated herpesvirus genome with the CRISPR-Cas9 platform in latently infected cells

**DOI:** 10.1186/s12985-021-01527-x

**Published:** 2021-03-17

**Authors:** Coral Orel Haddad, Inna Kalt, Yehuda Shovman, Lei Xia, Yehuda Schlesinger, Ronit Sarid, Oren Parnas

**Affiliations:** 1 The Mina and Everard Goodman Faculty of Life Sciences and Advanced Materials and Nanotechnology Institute, Bar-Ilan University, 5290002 Ramat-Gan, Israel; 2 The Concern Foundation at the Lautenberg Center for Immunology and Cancer Research, IMRIC, Hebrew University Faculty of Medicine, 91120 Jerusalem, Israel

**Keywords:** CRISPR-Cas9, Kaposi’s sarcoma-associated herpesvirus, KSHV, Latency associated nuclear antigen, LANA, open reading frame 73, orf73, open reading frame 45, orf45

## Abstract

**Background:**

Kaposi’s sarcoma-associated herpesvirus (KSHV) is a transforming gammaherpesvirus. Like other herpesviruses, KSHV infection is for life long and there is no treatment that can cure patients from the virus. In addition, there is an urgent need to target viral genes to study their role during the infection cycle. The CRISPR-Cas9 technology offers a means to target viral genomes and thus may offer a novel strategy for viral cure as well as for better understanding of the infection process. We evaluated the suitability of this platform for the targeting of KSHV.

**Methods:**

We have used the recombinat KSHV BAC16 genome, which contains an expression cassette encoding hygromycin-resistance and a GFP marker gene. Three genes were targeted: *gfp,* which serves as a marker for infection; *orf45* encoding a lytic viral protein; and *orf73,* encoding LANA which is crucial for latent infection. The fraction of cells expressing GFP, viral DNA levels and LANA expression were monitored and viral genomes were sequenced.

**Results:**

We found that KSHV episomes can be targeted by CRISPR-Cas9. Interestingly, the quantity of KSHV DNA declined, even when target sites were not functionally important for latency. In addition, we show that antibiotic selection, used to maintain infection, interferes with the outcome of targeting.

**Conclusions:**

Our study provides insights into the use of this fundamental approach for the study and manipulation of KSHV. It provides guidelines for the targeting CRISPR-Cas9 to the viral genome and for outcomes interpretation.

**Supplementary Information:**

The online version contains supplementary material available at 10.1186/s12985-021-01527-x.

## Introduction

The technology involving 'clustered regulatory interspaced short palindromic repeat' (CRISPR) is recognized as a revolution in the field of molecular biology research, gene editing, and gene therapy. In nature, the CRISPR-Cas9 system functions in bacteria and archaea as a barrier for foreign genetic elements and viruses that also creates acquired long term immunity. This system has been simplified and adapted for use in mammalian cells [1–3] and other organisms such as yeast and plants to enable efficient gene editing [4–6]. The technology involves the expression and targeting of Cas9 to DNA sequences by specifically designed single-guide RNAs (sgRNAs). In principle, it can target any sequence of choice, provided that it is followed by an NGG protospacer adjacent motif (PAM). Once Cas9 accesses its DNA target it generates double-stranded breaks (DSBs) that can be repaired via two main repair mechanisms: non-homologous end-joining (NHEJ), which generates imprecise insertion or deletion (INDEL) mutations that may cause frameshift or disrupt amino acid coding, and homologous repair (HR), involving a homologous DNA fragment as a repair template.

The CRISPR-Cas9 system has been successfully applied for editing, activating or repressing specific genes of interest in the context of a wide variety of genomes. This also includes viral genomes, in particular those of double-stranded (ds) DNA viruses that generate long term persistence such as Hepatitis B virus (HBV) and herpesviruses, as well as the Human Immunodeficiency Virus (HIV) provirus [7–9]. Accordingly, this methodology offers a new approach to antiviral therapy involving either direct targeting of viral genomes to impair their replication and to eliminate them from the host, or targeting of selected host genes that are necessary for virus infection and propagation. This technology can also be used for basic research investigating the biology of viral infections and for the manipulation of viral genomes towards the generation of new recombinant viruses, to enable identification of cellular and viral gene products that participate in the modulation of viral infection and its outcomes.

Kaposi’s sarcoma-associated herpesvirus (KSHV), also known as human herpesvirus 8 (HHV-8), is a cancer-related gamma-2 herpesvirus which is etiologically implicated in all types of Kaposi's sarcoma (KS). In addition, KSHV is the causative agent of other disorders, including primary effusion lymphoma (PEL), the plasmablastic variant of multicentric Castleman's disease, and KSHV-inflammatory cytokine syndrome [10–15]. In common with all other herpesviruses, KSHV infection may be either lytic or latent. Lytic infection is characterized by extensive expression of viral genes, replication of the viral genome, and assembly and release of new virions. In contrast, during latency, KSHV DNA exists as multiple circular episomes, tethered to host chromosomes in the nuclei of infected cells, from which it expresses a limited set of viral genes that enable long-term infection. Latency is largely regulated by the latency-associated nuclear antigen (LANA), encoded by *orf73,* which plays crucial roles in the maintenance, replication and segregation of KSHV episomes during mitosis. This gene product also alters cell transcription and signaling programs and suppresses lytic replication [16–19]. Accordingly, targeting of LANA by different methods has been previously reported to lessen KSHV episome maintenance [20–22]. Thus, LANA protein and its coding gene, *orf73*, are considered key targets for therapeutic modulation in KSHV-infected individuals who are either asymptomatic carriers, or present with a KSHV-related disease.

In the present study, we evaluated the suitability of the CRISPR-Cas9 platform for targeting the KSHV genome during latency. We found that latent KSHV episomes can be efficiently targeted by the CRISPR-Cas9 method and that targeting *orf73*, compared to targeting a non-functional gene (*gfp*), increases the loss of viral episome. In addition, we show that antibiotic selection to maintain latent KSHV infection phenotypically interferes with the outcome of the targeting of the KSHV episomes. Thus, we show that CRISPR-Cas9 is useful in targeting KSHV genome and therefore is an efficient tool for *cis* genetic targeting and functional research of viral genes and genomic elements. We provide insights regarding the use of this fundamental approach for the study and manipulation of KSHV infections.

## Materials and methods

### Cell culture and viruses

SLK cells (kindly provided by Prof. Don Ganem (University of California) and Prof. Rolf Renne (University of Florida)) [23] were maintained in Dulbecco’s modified Eagle’s medium (DMEM) supplemented with 10% fetal calf serum (FCS), 50 IU/ml penicillin and 50 µg/ml streptomycin (Biological Industries, Kibbutz Beit Haemek, Israel). These cells were transduced with a recombinant lentivirus encoding Cas9, selected with 7.5 µg/ml Blasticidin (A.G. Scientific Incorporation), and infected with a recombinant BAC16-based KSHV genome encoding N-terminal mCherry-tagged ORF45 which is expressed upon lytic induction [24]. Infected cells were selected and maintained with 600 µg/ml hygromycin (Sigma).

### Production of recombinant lentiviruses and transduction

Cas9 was expressed using the recombinant LentiCas9-Blast (Addgene 52962) [25]. Oligonucleotides with complementary sequences, listed in Table 1, were annealed in a thermocycler under the following conditions: 95 °C for 5 min, followed by ramp down to 25 °C at 5 °C/min. The resulting fragments were then cloned into BsmBI digested lentiGuide-Puro (Addgene #52963), and insertion was confirmed by sequencing. Lentiviral particles were produced in HEK-293T cells by calcium-phosphate transfection of the lentiviral expression vectors along with Gag-Pol-Rev and VSV-G packaging plasmids. Supernatants were collected 72-h post transfection and cell debris were removed by filtering through a 0.45-µm filter. 1 µg/ml Polybrene was added to the medium during transduction, and transduced cells were selected with 7.5 µg/ml Blasticidin for LentiCas9-Blast and with 1 µg/ml puromycin for sgRNA-encoding lentiviruses.

**Table 1:** *sgRNAs used, and their corresponding position within the target open reading frame.* Column 1: sgRNA name. Column 2: sgRNA nucleotide position in the orf. Column 3: sgRNA sequence

Designation	Position within open reading frame	Sequence
*gfp sgRNA 1*	329–348	GCGCCGAGGTGAAGTTCGAG
*gfp sgRNA 2*	563–582	CCATCGGCGACGGCCCCGTG
*orf45 sgRNA 1*	184–202	CCCAGACGACGTCTTCGCCG
*orf45 sgRNA 2*	253–272	GATCAGTCGAGCGGCGAATC
*orf73 sgRNA 1*	113–132	ACCTACATCTACAACCGCGA
*orf73 sgRNA 2*	143–162	TCGCCGACTCCGTCGACGGC

### Western blot analysis

Cells were washed twice in cold PBS, suspended in RIPA lysis buffer, and incubated on ice for 30 min. Cell debris were removed by centrifugation at 12,000 g for 15 min at 4 °C. Protein lysates were resolved by SDS-PAGE and transferred to nitrocellulose membranes using Trans blot turbo RTA midi nitrocellulose transfer kit (BioRad). The protein content of the samples was verified by Ponceau S staining. The nitrocellulose membranes were blocked with 5% dry milk in TBS, and subsequently incubated with primary mouse antibodies to GFP (Covance Research Products), LANA (MBL International Cooperation) and Tubulin (E7-S, DSHB). Specific reactive bands were detected using anti-mouse conjugated to horseradish peroxidase, and were visualized using Clarity Western ECL substrate (BioRad).

### FACS quantification of GFP-positive and negative cells

Cells were trypsinized, washed and suspended in 0.5 ml PBS. Next, 0.5 ml 4% formaldehyde was added, and the cells were incubated at 4 °C for 20 min. Cells were washed with PBS, and GFP-positive cells were determined by fluorescence-activated cell sorting (Gallios, Bekmanculter). Data analysis was performed using FlowJo software.

### Quantitative TaqMan real-time polymerase chain reaction

Total DNA was extracted by EZ-DNA Total DNA Isolation kit (Biological Industries, Israel). KSHV DNA was quantified using a TaqMan-based real-time PCR assay with FAM-labeled fluorescent *orfK6* primers (Sigma) along with the cellular Cy5-labeled ERV3 gene primers. The total volume of the PCR reaction was 20 μl, and the reaction was performed for 40 amplification cycles at 95 °C for 15 seconds and 60 °C for 45 seconds. Efficiency of the reaction was determined by the slope of a calibration curve. The average reaction efficiency was calculated, and the differences between mean values (Ct) of the *orfK6* gene were calculated. PCR reactions were run in triplicates on CFX96 Touch Real-Time PCR Detection system (Bio-Rad).

### Immunofluorescence microscopy

Cells were seeded on coverslips and were transduced with recombinant lentiviruses. After 16 days, cells were washed with PBS and fixed in 4% formaldehyde for 20 min at room temperature. The cells were then washed three times in PBS and permeabilized in PBS containing 0.2%TritonX-100 and 1% bovine serum albumin (BSA) at room temperature for 30 min. Cells were probed with primary rat antibody to LANA (LN53, Santa Cruz) at 40 °C, and a conjugated secondary anti-rat Alexa Fluor 647 (Jackson Labs) was then applied for detection. To stain the nuclei, the cells were incubated for 30 min with 0.05 µg/ml Hoechst dye (Sigma). Cells were examined and photographed under a confocal laser-scanning microscope (Leica STED Live Imaging).

### Sequencing

Two successive PCR reactions were performed using 100 ng DNA as a template. The first PCR was done using site specific primers, flanking the relevant sgRNA, and the second reaction was performed using barcoded primers that included Illumina adaptors (primers are listed in Table 1 and Table 2). To amplify the *gfp* fragment we used the 2nd forward primer, and to amplify *orf73* and *orf45* fragments we used the 1st forward primer. Sequencing was done using Nextseq500 platforms (one side 150 bp). After the sequencing data was demultiplexed, the adapter sequences were removed by Cutadapt (ver. 1.18), and the reads were aligned to either *gfp*, *orf45* or *orf73*. Primer dimers as well as reads with low quality were filtered out. The alignment was performed using swalign from the Bioinformatics Toolbox for Matlab (ver 9.7.0.1216025), and reads that did not contain the cutting site were removed. Insertions and deletions in the area of the cutting site were mapped, and the percentage of reads containing indels was calculated. The location of the deletions relative to the cutting site and the length of the deletions was also estimated.

**Table 2:** *Primers used for PCR amplification and sequencing.* Column 1: Gene name. Column 2: primers sequence. Column 3: PCR product length

Gene	Primer pair	*Size of PCR product
*gfp*	1st F: TCTTCTTCAAGGACGACGGC 2nd F: GCAGCTCGCCGACCACTAC 1st + 2nd R: TTACTTGTACAGCTCGTCCA	462 bp 226 bp
*orf73*	1st F: AGATGTGACCTTGGCGATG 2nd F: CAACCGCGAAGGAAGCAT 1st + 2nd R: CGGAGACACAGGATGGGATG	313 bp 282 bp

^*^Sizes ignore potential deletions and insertions due to single or double cut by Cas9

### Statistical analysis

Two-way ANOVA was performed to test the effect of CRISPR targeting on the fraction of cells expressing GFP and on the levels of KSHV DNA, followed by Tukey’s post-hoc pairwise analysis for all time points together.

## Results

To test the competence of the CRISPR-Cas9 platform for the targeting of the KSHV genome, we employed the human renal cell carcinoma SLK cell line which is widely used as a model system to study KSHV infection [23]. First we generated, by transduction of LentiCas9-Blast [25], SLK cells that constitutively express Cas9 under the control of the EF-1α promoter. After blasticidin selection, the resulting cells were infected with BAC16-mCherry-ORF45 recombinant KSHV, and selected with hygromycin. The BAC16-mCherry-ORF45 clone was previously generated based on the recombinant bacterial artificial chromosome 16 (BAC16) containing the entire KSHV genome and a GFP-IRES-hygromycin expression cassette under the control of the EF-1α promoter; it also encodes the immediate-early lytic gene product ORF45 protein fused at its N-terminus to monomeric Cherry fluorescent protein (mCherry) [24, 26]. BAC16-based recombinant KSHV can be manipulated in *E. coli* and has become a valuable tool to enable characterization of the different genes and functional domains of KSHV [26]. Following transfection of BAC16-mCherry-ORF45 into cells and hygromycin selection, the vast majority of the cells express GFP, which enables detection of infected cells, while expression of mCherry tracks cells undergoing lytic infection.

Three genes that are encoded within the recombinant viral genome were targeted using the CRISPR-Cas9 approach: (i) *gfp,* which serves as a marker for infection though it is not necessary for the maintenance of the KSHV episome nor during lytic replication; (ii) *orf45* encoding an immediate-early lytic viral protein which is not expressed in SLK cells during latent infection and is not needed for the maintenance of KSHV episomes in these cells, and (iii) *orf73* encoding the latency-associated nuclear antigen (LANA), which is required for the maintenance, replication and segregation of latent KSHV episomes. Each gene was targeted with two sgRNAs (Table 1) that were cloned and introduced into the cells using recombinant lentiviruses and transcribed under the control of the human U6 promoter. The use of two sgRNAs for each gene improves the editing efficiency in the described multi-episomal system as it allows higher indel frequency as well as large deletions. Untransduced cells and cells transduced with a non-targeting sgRNA were used as controls. Cells were maintained in the absence or presence of hygromycin selection, while cells transduced with the sgRNA encoding constructs were selected with Puromycin. Cells were trypsinized 6, 9, 13 and 16 days post transduction and collected. An aliquot containing 1/6 of the cells was replated and the harvested cells were examined for GFP expression and for the KSHV DNA burden.

### Analysis of GFP expression upon targeting of BAC16-mCherry-ORF45 by CRISPR-Cas9

**Fig. 1:**
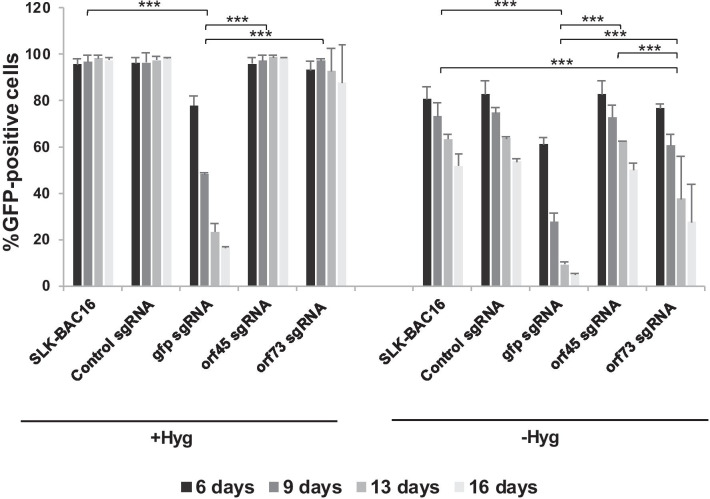
GFP expression in SLK-BAC16-mCherryORF45-infected cells following targeting of selected genes by CRISPR-Cas9. BAC16-mCherryORF45-infected SLK cells expressing Cas9 were transduced with recombinant lentiviruses encoding a random non-targeting sgRNA (control sgRNA) or sgRNAs targeting *gfp*, *orf45* and *orf73*. Each gene was targeted with a combination of two guides. Transduced cells were selected with 1 µg/ml puromycin and were grown in the absence or presence of 600 µg/ml hygromycin. Cells were harvested 6, 9, 13 and 16 days post transduction, and GFP expression was monitored by FACS analysis. Mock treated SLK-BAC16 cells that were either maintained in the presence or absence of hygromycin were used as controls. *p < 0.05, **p < 0.01, ***p < 0.001. Statistical test was performed between groups including all time points together

**Fig. 2:**
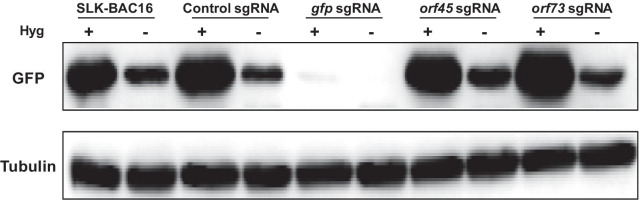
Western blot analysis of GFP in SLK-BAC16-mCherry-ORF45-infected cells 16 days following targeting of selected genes by CRISPR-Cas9. BAC16-mCherry-ORF45-infected SLK cells expressing Cas9 were treated as described in Fig. 1. On day 16 post transduction, cells were collected, and protein extracts were prepared. Samples containing 40 µg of protein were used to determine GFP protein expression; anti-Tubulin was used as a loading control

FACS analysis was used to determine the fraction of cells expressing GFP, which is encoded by the recombinant BAC16-mCherryORF45 KSHV genome (Fig. 1). In line with previous reports [27, 28], spontaneous loss of GFP expression was evident in cells that were grown in the absence of hygromycin selection, and cells grown in the absence of selection demonstrated a higher degree of GFP loss under all assay conditions as compared with the corresponding cells that were maintained with hygromycin. As expected, targeting of *gfp* resulted in a dramatic decrease in the fraction of cells expressing GFP, that became more prominent over time. The fraction of cells expressing GFP was significantly lower in cells that were transduced with sgRNAs targeting *gfp* (both in the presence or absence of hygromycin selection) as compared with control non-transduced cells, and cells that were transduced with a control non-targeting sgRNA, *orf45* sgRNAs and *orf73* sgRNAs. Targeting of *orf45* did not affect the expression of GFP in either cells that were maintained with hygromycin selection nor in the absence of hygromycin. Accordingly, the fraction of cells expressing GFP was not significantly different in *orf45* sgRNA transduced cells as compared with untransduced cells, and cells that were transduced with non-targeting control sgRNA. Targeting of *orf73* resulted in a moderate, but highly significant, decrease of GFP expression that was evident only in cells grown in the absence of hygromycin selection (Fig. 1). Of note, extensive cell death, in particular at 9 and 13 days post transduction, was observed in cells that were transduced with *orf73* sgRNAs and maintained with hygromycin selection (not shown), suggesting the occurrence of episome loss, which was coupled with loss of hygromycin resistance. This result suggests that our experimental setting can be used for KSHV targeting and in addition, that inclusion of hygromycin in the growth medium imposes selection towards cells that maintain the expression of the *orf73* protein product, LANA. These finding was further supported upon analysis of median fluorescence intensity of the GFP-positive cells (Additional file 1: Fig. 1). A large reduction in the median fluorescence intensity was evident in cells that were transduced with *gfp* sgRNA and maintained with or with no hygromycin selection, whereas a gradual decrease in GFP intensity was evident in control cells and cells transduced with the other sgRNAs and grown with no hygromycin selection. Targeting of *gfp* in the presence of hygromycin selection resulted in a significant decrease in fluorescence intensity in comparison with the other sgRNAs and control. The intensity of GFP was not significantly different upon targeting of this gene in the presence or absence of selection and it was largely decreased. In line with this result, western blot (WB) analysis of protein extracts from cells that were harvested 16 days after the transduction demonstrated similar levels of GFP expression in all cells that were maintained in the presence of hygromycin selection except for cells that were transduced with sgRNAs targeting *gfp* (Fig. 2). Expression of GFP was lower in cells that were maintained in medium lacking hygromycin. Of note, GFP levels were slightly higher in cells that were transduced with *orf73* sgRNAs that were maintained with hygromycin, probably due to selection of cells containing higher copies of the viral episome of which a fraction may encode non-functional LANA-1.

### Quantitative analysis of KSHV DNA

**Fig. 3:**
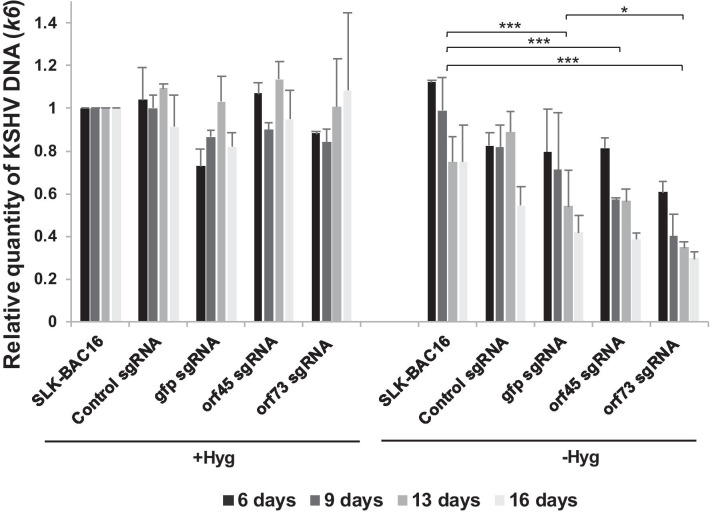
Viral DNA quantification following CRISPR-Cas9 targeting of *gfp*, *orf45* or *orf73*. BAC16-mCherry-ORF45-infected SLK cells expressing Cas9 were treated as described in Fig. 1. DNA was extracted and subjected to TaqMan real-time PCR for viral DNA using primers that target the viral *orfK6* gene along with the cellular *erv-1* gene, which was used to normalize loading. Statistical test was performed between groups including all time points together for each gRNA treatment. *p < 0.05, ***p < 0.001

**Fig. 4:**
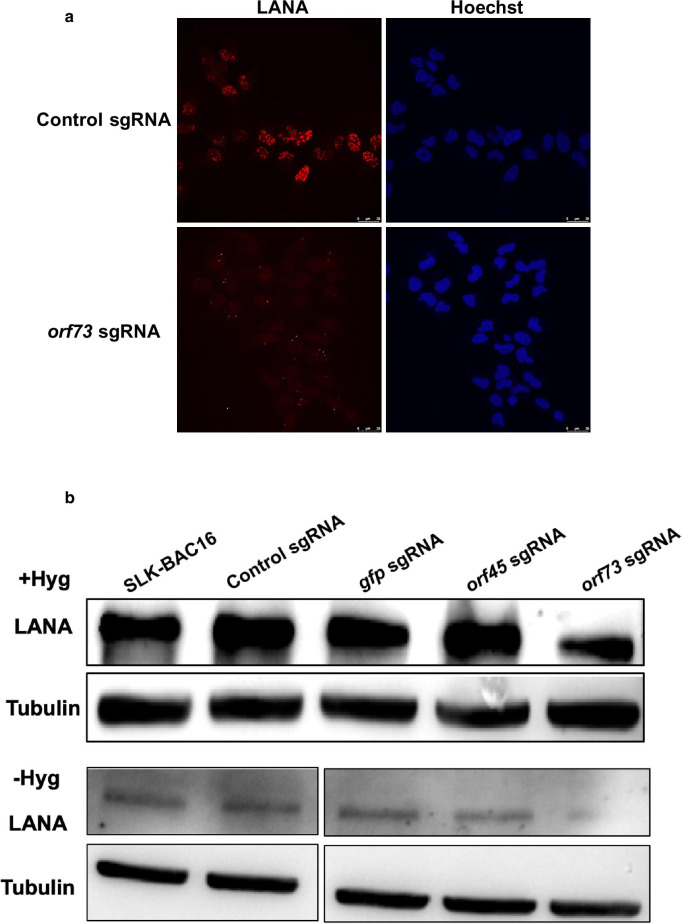
Visualization of LANA dots and Western blot analysis of LANA 16 days following targeting of *orf73* by CRISPR-Cas9. Cas9-BAC16-mCherryORF45-infected SLK cells were transduced with recombinant lentiviruses expressing a random non-targeting sgRNA or *orf73* sgRNAs. Cells were maintained with no hygromycin selection, fixed 16 days post transduction and stained with rat monoclonal anti-LANA followed by anti-rat Alexa Fluor 647-conjugated secondary antibodies. The corresponding staining of nuclear DNA by Hoechst is also displayed (**a**). BAC16-mCherryORF45-infected SLK cells expressing Cas9 were treated as described in Fig. 1. Cells were collected 16 days post transduction, and protein extracts were prepared. Samples containing 60 µg of protein extracts from cells that were maintained with hygromycin and with no hygromycin selection, respectively, were used to determine LANA protein expression while Tubulin was used as loading control (**b**)

To study the effect of gene targeting on the burden of KSHV DNA, we extracted DNA from cells at each time point and subjected it to TaqMan real-time PCR assay. As shown in Fig. 3, the quantity of the KSHV DNA in cells that were grown with hygromycin was not significantly affected by transduction of the different sgRNAs at any time point. This supports the notion that the addition of hygromycin to the growth media enables selection of cells that harbor a threshold quantity of KSHV episomes. In contrast, targeting of *gfp* in cells that were maintained in the absence of hygromycin produced a significant decrease in the quantity of KSHV DNA as compared with control non-transduced cells. The quantity of KSHV DNA was not significantly different upon targeting of *gfp* as compared with *orf45*. GFP expression was used as a marker for infection in the context of BAC16, yet neither this gene nor its product are necessary for the maintenance of KSHV episome. Similar results were obtained upon targeting of *orf45,* an immediate-early lytic gene, which is not expressed during latent infection and does not have a role during latency in SLK cells [24, 29–31]. Therefore, it appears that the reduction in the burden of KSHV DNA upon targeting of these genes is due to incomplete repair of cleaved DNA, and is not associated with the functions of the protein products of these genes. Targeting of *orf73* generated a significantly larger decline in the quantity of KSHV DNA as compared with *gfp* (*p* = 0.015) and *orf45* (*p* = 0.052) targeting, suggesting that the reduction of KSHV DNA upon targeting of *orf73* is due to both incomplete repair and the unique and essential functions of LANA for the maintenance of latent KSHV infection. Loss of KSHV episomes upon targeting of LANA was confirmed by evaluating the number of LANA dots in the nucleus, which was previously shown to correlate with the number of KSHV episomes [27, 32]. As shown in Fig. 4, cells that were transduced with *orf73* sgRNAs and maintained without hygromycin selection exhibited a significant reduction in the number of LANA dots per nuclei. Therefore, our data indicate that when investigating the function of latent genes hygromycin selection is not recommended. These findings were further supported by WB analysis of protein extracts from cells that were harvested 16 days after the transduction. As shown in Fig. 4B, LANA was detected in all cell lysates, yet the intensity of the bands was weaker in cells that were transduced with sgRNAs targeting *orf73*.

### Sequence analysis

**Fig. 5:**
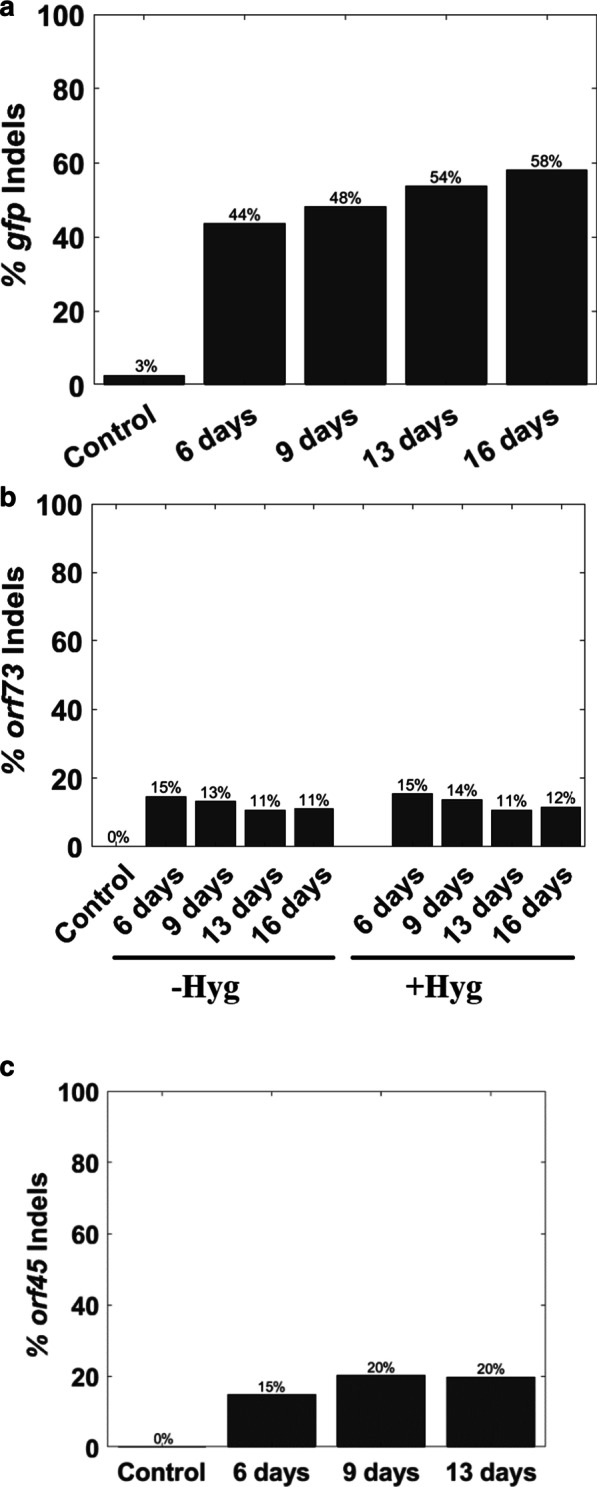
Sequencing the episome CRISPR-Cas9 cut sites. To map insertions and deletions (indels), we amplified genomic DNA and subjected it to deep sequencing. The percentage on the y axis represents the total number of reads with indels divided by the total numbers of reads. The identities of the samples are indicated on the x axis, including the name of the gene and the number of days post sgRNA transduction. Percentage of indels flanking the cutting site in *gfp* using the hygromycin minus sgGFP samples (**a**). Percentage of indels flanking the cutting site in *orf73* using both hygromycin minus and hygromycin plus samples (**b**). Percentage of indels around the cutting site in *orf45* using hygromycin minus samples (**c**)

To assess whether the CRISPR-Cas9 achieved editing of the viral genome at the corresponding sgRNA targets, we deep sequenced PCR amplicons from each time point. As shown in Fig. 5, the *gfp* sgRNA target sites demonstrated a relatively high indel mutation frequency that increased over time. This result is consistent with our FACS analysis that demonstrated gradual reduction in the percentage of GFP-positive cells. Lower indel frequency was evident in *orf45* and *orf73* PCR products and the indel level did not increase over time. Similar results were obtained in the presence or absence of hygromycin-selection upon targeting *orf73* (Fig. 5).

**Fig. 6:**
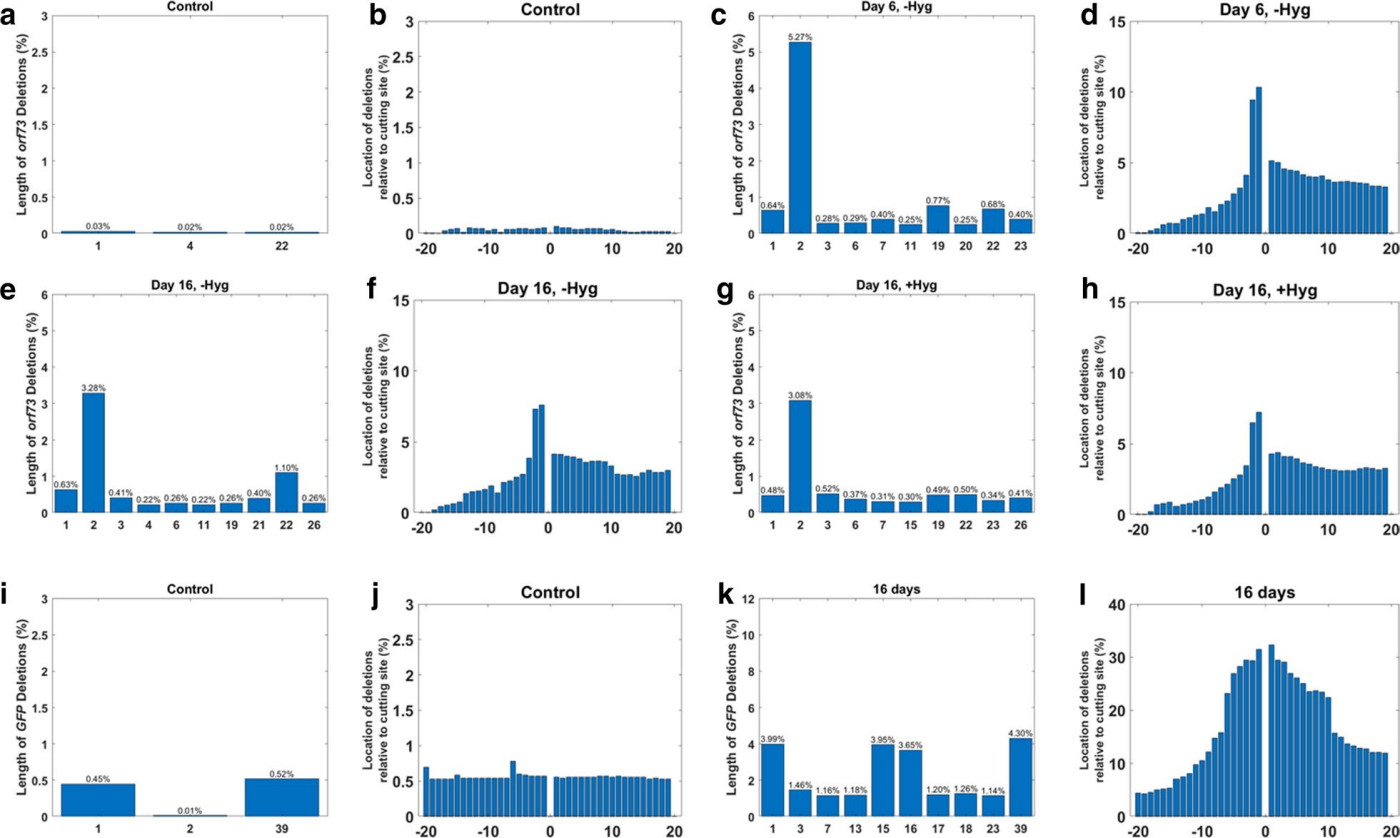
Distribution and abundance of deletions flanking CRISPR-Cas9 cut sites. The graphs show the percentage of nucleotide deletion based on amplicon deep sequencing. Cutting site in *orf73* (**a**–**h**), cutting site in *gfp* hygromycin minus (**i**–**l**) and no guide was used (**a**, **b**, **i**, **j**) (control). (**a**, **c**, **e**, **g**, **i**, **k**) the graphs show the most abundant deletion length. (**b**, **d**, **f**, **h**, **j, l**) the graph shows the distribution of deletions around the cutting site (0). Time points are indicated in the title. **a**, **c**, **e**, **g**, **i**, **k** the x-axis represent the length of the most highly frequent deletions. **b**, **d**, **f**, **h**, **j**, **l** the x-axis represent the frequency of a base-pair to be deleted, in both sides of the cutting cite (0- between nucleotides 3 and 4 5′ to the pam sequence

We next analyzed the indels pattern using the deep sequencing data (Fig. 6). We could observe that the indel distribution was consistent for each guide in all the corresponding samples. For example, two nucleotide deletion in *orf73* was the most abundant indel in all time points with and without hygromycin selection. This out-of-frame mutation, in a latent essential gene, may point that the surviving cells include a mixture of edited and non-edited episomes. The indel distribution of *gfp* targeted cells included dominant deletions in the length of 1,15, and 16 base-pairs which was different from the deletion pattern in *orf73* gene, as shown in Fig. 6.

The results presented in Fig. 5 and 6, indicates that cells expressing non-functional mutated LANA protein, lose the KSHV episomes and are either eliminated by hygromycin selection or continue to grow with no KSHV infection in the absence of selection. However, in some cases a mixture of episomes in the same cells may be sufficient to produce enough LANA for episome maintenance.

## Discussion

Although relatively new, the CRISPR-Cas9 platform has already been extensively used to reveal the involvement of cellular proteins in the control of infection pathways in a variety of viruses [33]. By using different cell models, these screens identified involvement of the cellular gene products MCLI and XPOI in KSHV-related tumorigenesis, as well as host-dependency factors that are critical for EBV-transformed B cells [34–36]. In addition, direct manipulation of several herpesvirus genomes using the CRISPR-Cas9 platform was reported [7, 33, 37–39]. Furthermore, the effectiveness of CRISPR-Cas9 in eliminating latent EBV episomes in cell culture, by targeting key factors for latent genome replication, has been described [37, 40]. Finally, a significant reduction of KSHV episome burden in latently infected epithelial Vero219 and endothelial LIT2 cells following a single transduction of replication-incompetent adenovirus encoding Cas9 and sgRNA targeting *orf73* was recently reported [41].

The present study provides several guidelines for the use of the CRISPR-Cas9 approach for the manipulation of KSHV genome and for the study KSHV biology. These guidelines could hold true for other viruses, as well. Targeting the viral genome can be used for cellular cure and episome lose, or for gene editing for functional studies. However, these two main applications may contradict, as episome loss can eliminate efficient latent essential gene knockout. The detailed time course profiling of episome lose and gene editing with and without hygromycin selection in our study, shows that under different conditions both can be achieved in the same system. Without hygromycin selection, targeting of latent essential gene, *orf73*, results in reduction of viral DNA load and such reduction is dominant compared to viral DNA load reduction following targeting of non-essential genes such as *gfp* and *orf45*. In addition, our study suggests that in the same system efficient gene knockout of non-essential genes can be achieved. Targeting of *gfp,* which is not necessary for latency, reduces GFP level while maintaining similar viral load, in the presence of hygromycin selection compared to non-targeting guide. Thus, non-essential gene targeting is efficient in our setting and overcomes episome loss indicating that efficient gene knockout is possible in the exact same system if selection is used. This finding does not suggest that episomes are resistance to DSB and that DNA repair is perfect, as in the absence of hygromycin selection the reduction in GFP signal was stronger, but rather that the cells compensate temporal episome lose and that episome number is tightly controlled. Therefore, intact episome number with gene knockout can be maintained over time. This can be used for the recovery of Bacmids containing specific mutations or for exploring the function of genes that are not involve in maintenance of latency. Our setting is also suitable for the inspection of genes that play a role in maintenance of latency, such as *orf73*. In that case, we can observe strong phenotype without hygromycin, as we see reduction in viral DNA load. In the presence of hygromycin the phenotype is weak and cells overcome this condition in yet unknown mechanism. It is possible that certain viral episomes evade CRISPR-Cas9 as a result of a unique chromatin organization or nuclear localization or that cleavage enabled evasion from further targeting yet did not affect the function of LANA. Furthermore, as cells carry multi episomes it could be that episomes encoding non-functional LANA are maintained via LANA which is expressed in trans from other episomes.

Our study suggests the ongoing targeting of the KSHV genome during the course of the experiment. This could be due to the multiple number of KSHV episomes in latently infected cells, which challenge the CRISPR-Cas9 platform. Of note, proteins with long half-life, such as GFP and LANA, could remain in the cells for relatively long periods, and thus phenotypic screens should involve relatively long-term follow up. In addition, our study suggests that functional screens of KSHV using the CRISPR-Cas9 platform should not utilize antibiotic selection of KSHV episomes. This is clearly shown when *orf73*, which is known to be essential for the maintenance of latency, was targeted. In fact, we noticed a large decrease in the number of viable cells, in the presence of hygromycin, upon targeting of *orf73* at 9–13 days following sgRNA transduction (data not shown). Yet, FACS analysis of GFP positive cells and WB analysis with anti-GFP did not identify significant changes in the fraction of cells expressing GFP upon targeting of *orf73* in the presence of hygromycin selection; nevertheless, KSHV DNA loads slightly decreased with time.

Targeting of *gfp* and *orf45,* which are not required for the maintenance of KSHV episomes, resulted in a time-dependent gradual decrease in KSHV DNA loads in cells that were maintained in the absence of hygromycin. This suggests that the repair of cleaved KSHV DNA is incomplete, and that a fraction of KSHV episomes are eventually degraded and lost regardless of the target sequence. Accordingly, caution must be taken when performing functional studies that target the viral genome, since the reduction of viral DNA load could result from incomplete repair rather than indicating a specific functional role of the targeted sequence. In fact, successful knockout of the *orf57* lytic gene from all copies of KSHV genome in PEL cells has been recently achieved, yet unexpectedly, it led to reduction of viral genome copies, likely due to incomplete repair [42]. Our study employed BAC16-mCherryORF45-infected cells, which enable continuous tracking of induction of lytic replication through microscopic detection of the immediate-early lytic protein mCherryORF45 [24]. Importantly, we did not detect any change in the number of mCherryORF45-positive cells under any experimental conditions, suggesting that the cleavage of the KSHV episome by Cas9 does not induce signaling pathways that promote lytic virus reactivation. Finally, the indel mutations suggest that the CRISPR-Cas9 platform offers an efficient approach for the manipulation of KSHV genome compared to in vitro targeting of BAC genome which is more labor intensive. The profiling of indel mutations in the sgRNAs predicted cutting sites in the *orf73* and *gfp* coding regions, shows that functional gene targeting and genetic manipulation of KSHV coding and non-coding genes can be done systematically to produce a collection of mutated episomes.

Systematic targeting of the viral genome will assist in finding the most highly efficient guides, that induce complete knock-out of essential and non-essential genes, and can therefore increase episome lose and viral episome elimination. Our system also can help to explore the minimal intact copy number of *orf73* that support episome maintenance, if single cell approach is used.

In summary, our experimental setting allows targeting of latent non-essential genes, and in this case, hygromycin selection is recommended. To test if a certain gene is essential or not essential for latent infection, growing the cells without hygromycin selection is favorable. Finally, targeting genes that maintain latent infection and growing the cells without selection is most efficient for latent viral elimination and cell curing.

## Conclusions

Our results provide evidence that targeting genes that play a role in maintenance of latency may cure cells from herpesvirus infection, and therefore could have therapeutic potential if delivery issues and off-target activity are solved. In addition, we show that KSHV genome editing is possible and can be an effective tool to target the viral genome with the kinetics described above; yet, we note that targeting any sequence in the viral genome may reduce viral genome copies probably due to incomplete repair. Further experiments will enable identification of the repair mechanism taking place during infection, and episome editing heterogeneity within each cell. Furthermore, this system can support genome-wide screens to identify host and viral genes that regulate KSHV infection. Finally, a similar approach can be used to target RNA viruses such as SARS-CoV-2 using appropriate CRISPR systems that target RNA.

## Supplementary Information

**Additional file 1** The median fluorescent intensity of GFP in SLK-BAC16-mCherryORF45-infected cells following targeting of selected genes by CRISPR-Cas9. The data is based on the experiments shown in Figure 1. On the Y-axis median florescent intensity, on the X-axis the different sgRNA samples at each time point, with and without hygromycin selection. Statistical test was performed between groups including all time points together. *p<0.05

## Data Availability

Upon acceptance all sequencing data will be deposited in Geo.

## Abbreviations

CRISPR: Clustered regulatory interspaced short palindromic repeat
sgRNAs: Single-guide RNAs
PAM: Protospacer adjacent motif
DSB: Double-stranded break
NHEJ: Non-homologous end-joining
INDEL: Insertion or deletion
HR: Homologous recombination
ds: Double-stranded
HBV: Hepatitis B virus
KSHV: Kaposi’s sarcoma-associated herpesvirus
HHV-8: Human herpesvirus 8
PEL: Primary effusion lymphoma
LANA: Latency-associated nuclear antigen
BAC: Bacterial artificial chromosome
WB: Western blot
DMEM: Dulbecco’s modified Eagle’s medium
FCS: Fetal calf serum
BSA: Bovine serum albumin
