# Supplementary figures and images for: Targeting the Kaposi’s sarcoma-associated herpesvirus genome with the CRISPR-Cas9 platform in latently infected cells
Source: Virol J. 2021 Mar 17;18:56. doi: 10.1186/s12985-021-01527-x (PMC7966637; doi:10.1186/s12985-021-01527-x)

**Supplementary Figure 1**


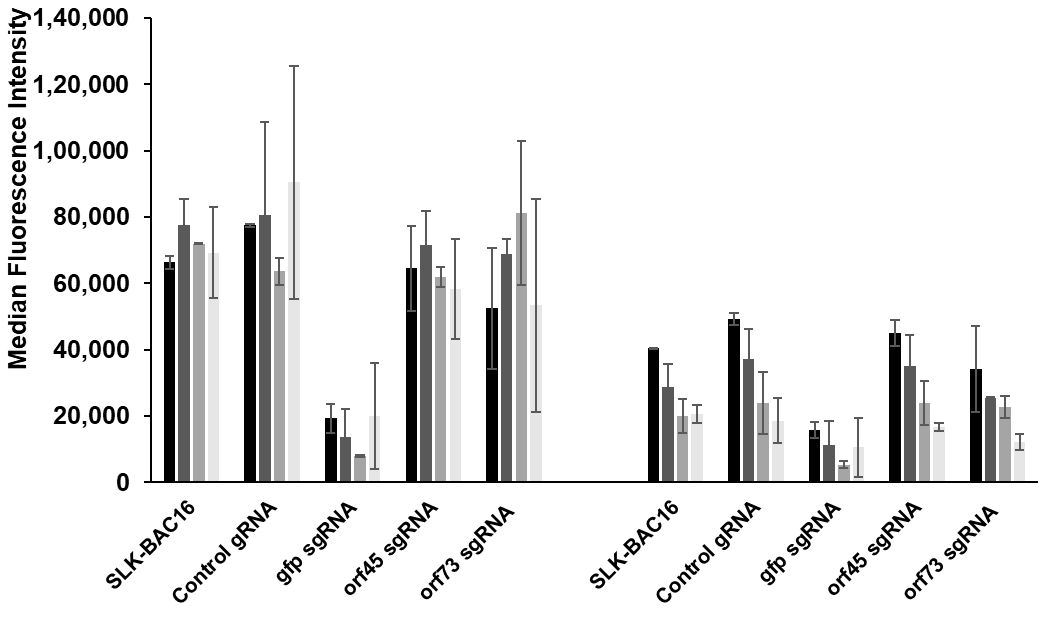


**+Hyg**

**-Hyg**

*

*

*

*

*

Supplement: Supplementary file 1 — Additional file 1 The median fluorescent intensity of GFP in SLK-BAC16-mCherryORF45-infected cells following targeting of selected genes by CRISPR-Cas9. The data is based on the experiments shown in Figure 1. On the Y-axis median florescent intensity, on the X-axis the different sgRNA samples at each time point, with and without hygromycin selection. Statistical test was performed between groups including all time points together. *p<0.05 [file 12985_2021_1527_MOESM1_ESM.docx]
